# 16S rRNA Gene PCR/Sequencing of Cerebrospinal Fluid in the Diagnosis of Post-operative Meningitis

**DOI:** 10.1099/acmi.0.000100

**Published:** 2020-02-03

**Authors:** Jahanavi M. Ramakrishna, Claudia R. Libertin, Joshua N. Yang, Mark A. Diaz, Anne L. Nengue, Robin Patel

**Affiliations:** ^1^​ Division of Infectious Diseases, Mayo Clinic, FL, USA; ^2^​ Division of Clinical Microbiology, Mayo Clinic, MN, USA; ^3^​ Division of Infectious Diseases, Mayo Clinic, MN, USA

**Keywords:** post-operative meningitis, aseptic meningitis, 16S rRNA gene, PCR, sequencing, molecular diagnosis

## Abstract

**Introduction:**

Post-operative meningitis (POM) is a life-threatening complication of neurosurgery. Diagnosis is often difficult due to pre-existing inflammation and antecedent antimicrobial use. Bacterial cerebrospinal fluid (CSF) cultures may reveal no growth, but empiric antibiotics are typically given due to the high morbidity and mortality associated with POM. 16S rRNA gene PCR/sequencing is a molecular methodology that can identify the presence of bacteria regardless of viability for culture.

**Case Presentation:**

A patient presented with a rapid onset of fever associated with headache, neck pain, nausea and altered mental status 11 days after undergoing laser interstitial thermal therapy for treatment of recurrent astrocytoma at another hospital. Based on clinical presentation and imaging, POM was suspected, and empiric antibacterial therapy was started. Microbiological stains and cultures of CSF were negative. Due to persistent fevers, 16S rRNA gene PCR/sequencing was done on CSF; it detected a member of the order *Enterobacteriales* most closely resembling *Serratia* species. All antimicrobials were stopped except for cefepime, which was given for 2 weeks. The patient’s mental status fully recovered.

**Conclusion:**

The application of 16S rRNA gene PCR/sequencing in the setting of POM is of value by improving the quality of patient care and decreasing costs by antimicrobial de-escalation. Further studies regarding the positive and negative predictive values of this test are required.

## Introduction

Meningitis following neurosurgery is a rare but potentially fatal condition [1]. Meningeal irritation can occur due to local inflammation (reaction to blood lysis, sutures, tissue breakdown or chemicals) or perioperative bacterial inoculation. Determining the cause of post-operative meningitis (POM) is crucial to prevent permanent neurological sequelae or death because the course of treatment for local inflammation varies from that of infection [2–5]. Practitioners often pre-emptively start empirical antimicrobial therapy before collecting cerebrospinal fluid (CSF) for diagnostic testing due to the high morbidity and mortality of POM. Antecedent antimicrobial use can inhibit bacterial growth, rending cultures negative [6]. We report a case of POM where 16S rRNA gene PCR/sequencing was used to identify a member of the order *Enterobacterales* most closely resembling *Serratia* species as the causative agent of POM in the presence of negative cultures, changing the antimicrobial management of the patient.

## Case Summary

A patient presented with altered mental status, fever, headache and neck pain. The patient had undergone laser interstitial thermal therapy for recurrent left frontal lobe astrocytoma at a local community hospital. The perioperative period was unremarkable. On post-operative day 11, the patient experienced confusion and nausea followed by rapid onset of fever, headache and neck pain.

Mental status deteriorated rapidly, and the patient was unable to provide a history. On examination, lethargy, dehydration and morbid obesity were noted. The temperature was 38.1 °C, and blood pressure was elevated at 171/93 mmHg. The patient’s surgical frontal surgical wound was healing without drainage. The patient was disoriented with regard to time, place and person. The patient's Glasgow Coma Score (GCS) was 10. The remainder of the physical examination was unremarkable. The patient was placed on respiratory support for airway protection and was admitted to the intensive care unit.

The peripheral leukocyte count was high (24.4×10^9^ l^−1^). Due to the possibility of POM, broad-spectrum intravenous antibiotics (cefepime, metronidazole and vancomycin) were started. Then, a lumbar puncture was performed, showing a CSF opening pressure of 11 cmH_2_O, 8300 leukocytes µl^−3^, and protein and glucose at 692 and 42 mg dl^−1^, respectively. CSF, blood and sputum cultures were without bacterial growth. The BioFire FilmArray Meningitis/Encephalitis multiplex PCR (bioMérieux) was negative. A QuantiFERON-TB Gold assay (Qiagen), performed due to a contact exposure history, was negative.

The patient remained obtunded and had persistent fevers to 39 °C. The infectious diseases division was consulted to determine the aetiology of the fever and to provide advice on antimicrobial utilization in the context of negative CSF cultures. No other potential source of infection was identified. Therefore, 16S rRNA gene PCR/sequencing was performed on the CSF. CSF was lysed with proteinase K and incubated with 0.1 mm silica beads in a thermomixer at 100 °C with rapid mixing; DNA was extracted with the Genomic DNA Clean and Concentrator 10 kit (Zymo Research). PCR was performed using the primers described by Gomez *et al*. and Virk *et al*. [7, 8] on a Roche LightCycler 480 instrument (Roche Molecular Systems) with SYBR Green detection. The PCR target, an ~400 bp portion of the V3–V4 region of the 16S rRNA gene, was bi-directionally sequenced and the sequence was analysed using SmartGene's Integrated Database Network System (www.smartgene.com). The sequence was 99.04, 99.04, 98.80 and 98.07 % identical to that of *Serratia nematodiphila*, *Serratia urealytica*, *Serratia marcescens* and *Serratia rubidaea*, respectively.

Antimicrobials were de-escalated to intravenous cefepime (2 g q12h) alone for 2 weeks. The fever resolved, and the patient cognitively improved to a GCS of 15. No recurrence of meningitis occurred over a follow-up of 7 months.

## Discussion

**Fig. 1.**
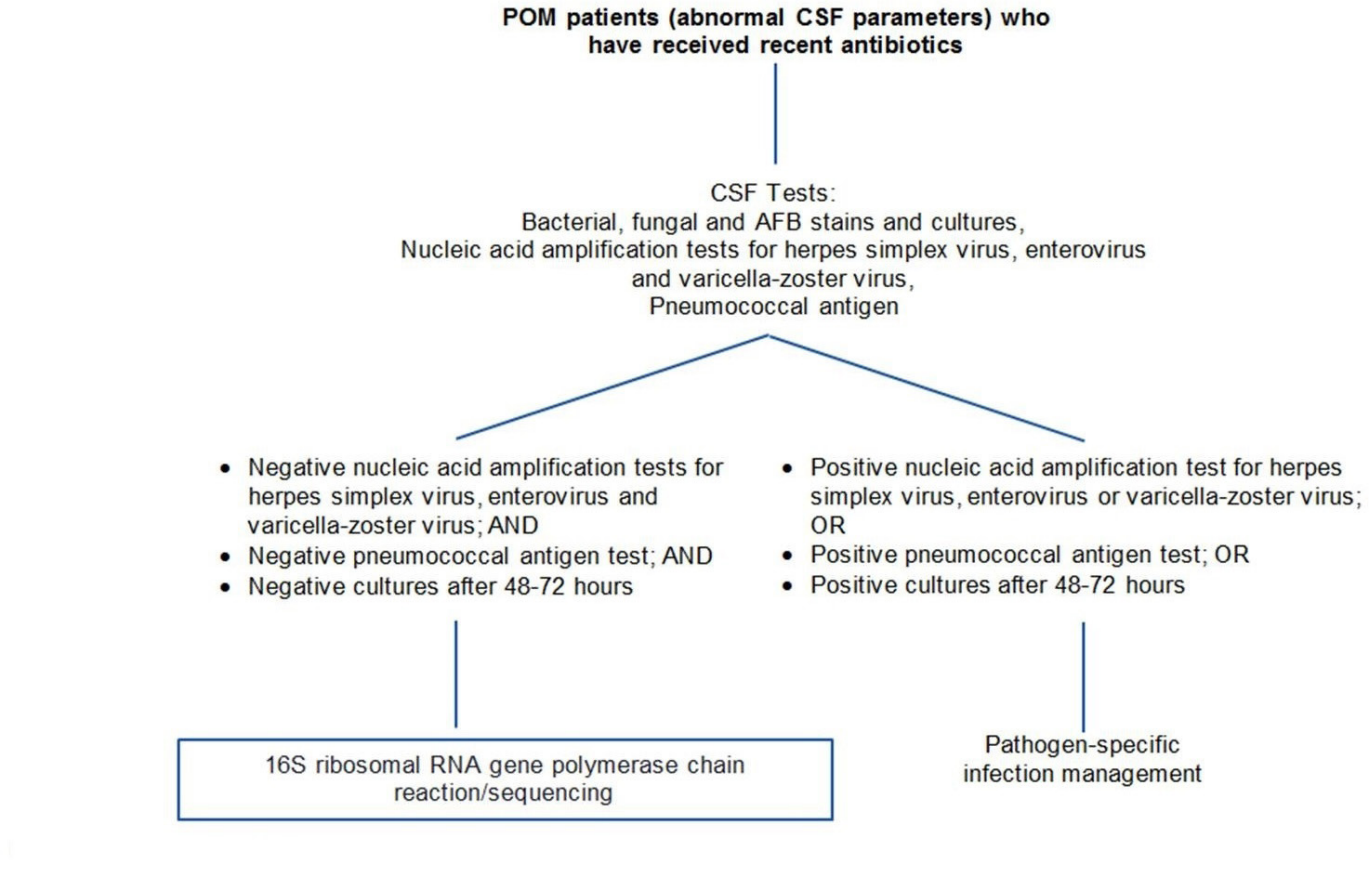
POM diagnostic algorithm. 16S rRNA gene PCR/sequencing is a molecular diagnostic approach that can add value to the laboratory diagnosis of POM. By informing de-escalation of antimicrobial agents, 16S rRNA gene PCR/sequencing is a test to be entertained in complex post-neurosurgical cases.

The diagnosis and management of POM pose a challenge because 70 % of cases are culture-negative [1, 9]. Bacterial meningitis remains a cause of poor neurological outcomes despite advancements in antimicrobial drugs and neuroscience [1]. As in this case, antimicrobial initiation may precede the acquisition of CSF for cultures. Early identification of the pathogen enables clinicians to make deliberate decisions regarding antibiotic therapy, reducing unnecessary antibiotic use and improving patient prognosis [5, 10, 11]. Stopping antimicrobials after 3–5 days as some studies have suggested, based on no growth of cultures, may not always lead to favourable clinical outcomes [9]. The current POM diagnostic ‘gold standard’ of CSF culture has limitations, including (i) the bacterial pathogenic mechanism relying on a small intraoperative bacterial inoculum, (ii) frequent antecedent antimicrobial exposure, (iii) the time-period required for bacterial growth in culture and (iv) deference of lumbar puncture in critically ill patients at some institutions [1, 11]. Even in situations when the lumbar puncture is performed before antibiotic administration, 15–30 % of CSF cultures may show no bacterial growth despite being the source of bacterial disease [12]. The management of post-neurosurgical meningitis in terms of the duration of antibiotic treatment for patients without microbiological confirmation infection is challenging [13]. Here we present (Fig. 1) a POM diagnostic algorithm that could potentially help providers balance existing limitations in diagnosing bacterial meningitis, suggesting when using 16S rRNA gene PCR/sequencing might be most helpful. The real-time 16S rRNA gene PCR/sequencing R methodology used here can be a useful diagnostic tool in culture-negative meningitis [11, 12, 14]. Because molecular analyses are not as affected by antimicrobials as is culture, they may have better predictive values than bacterial cultures. Targeting 16S rRNA allows clinicians to detect a broad range of bacteria, including non-cultivatable bacteria, as well as slow-growing bacteria such as mycobacteria that can be detected more quickly than culture [14].

Other studies have examined 16S rRNA gene PCR/sequencing to detect bacteria in CSF [15–17]. The application of this methodology in our case highlights its use in the setting of POM. A systematic review and meta-analysis of 16S rRNA gene PCR/sequencing in bacterial meningitis (14 studies, *n*=2780) showed the pooled sensitivity to be 92%, and pooled specificity to be 94 %. Because the meta-analysis also showed a high positive likelihood ratio (95%) and a low negative likelihood ratio (95%), a 16S rRNA gene PCR/sequencing test may be useful to exclude bacterial meningitis. This approach could enable physicians to better practice antimicrobial stewardship. The Infectious Diseases Society of America’s 2017 *Clinical Practice Guidelines for Healthcare-Associated Ventriculitis and Meningitis* calls for more studies to assess the negative predictive value of 16S rRNA gene PCR/sequencing in the setting of nosocomial aseptic meningitis [18]. In our case, a positive result detected a bacterium and prevented the discontinuation of antimicrobials in the face of a negative bacterial culture [11].

PCR is extremely sensitive, and detection of normal microbiota contaminating the specimen or cross-contamination of microbial DNA or amplified DNA can result in false-positive test results. Likewise, falsely negative results may occur due to inhibitors; this may be overcome by appropriate DNA purification steps before PCR [17, 19, 20]. False-negative results may also occur due to bacterial amounts present below the limit of detection of the assay. Studies using 16S rRNA gene PCR/sequencing of CSF report variability in sensitivity, possibly due to methodological differences. Despite these drawbacks, 16S rRNA gene PCR/sequencing is a valuable option in the diagnosis of POM. In the setting of no bacterial growth on culture after 48–72 h despite abnormal CSF parameters (at least 11 white blood cells mm^–3^, glucose <10 mg dl^−1^, lactate >4 mmol l^−1^), clinicians may consider 16S rRNA gene PCR/sequencing as the next best step when suspecting POM [18].
